# Drivers of groundwater utilization in water-limited rice production systems in Nepal

**DOI:** 10.1080/02508060.2019.1708172

**Published:** 2020-02-03

**Authors:** Anton Urfels, Andrew J. McDonald, Timothy J. Krupnik, Pieter R. van Oel

**Affiliations:** a Sustainable Intensification Program, International Maize and Wheat Improvement Center, South Asia Regional Office, Kathmandu, Nepal; b School of Integrative Plant Sciences, Soil and Crop Sciences Section, Cornell University, Ithaca, NY, USA; c Sustainable Intensification Program, International Maize and Wheat Improvement Center, Dhaka, Bangladesh; d Water Resources Management Group, Wageningen University, Netherlands

**Keywords:** Groundwater irrigation, decision processes, smallholders, resilience, Eastern Gangetic Plains, Nepal

## Abstract

Most rice farmers in Nepal’s Terai region do not fully utilize irrigation during breaks in monsoon rainfall. This leads to yield losses despite abundant groundwater resources and ongoing expansion of diesel pumps and tubewell infrastructure. We investigate this puzzle by characterizing delay factors governing tubewell irrigation across wealth and precipitation gradients. After the decision to irrigate, different factors delay irrigation by roughly one week. While more sustainable and inexpensive energy for pumping may eventually catalyze transformative change, we identify near-term interventions that may increase rice farmers’ resilience to water stress in smallholder-dominated farming communities based on prevailing types of irrigation infrastructure.

## Introduction

Feeding a projected global population of 9 billion in 2050 will require focused efforts to address trade-offs and capitalize on synergies between natural resources management and food security objectives, necessitating broad-based transitions to sustainable intensification technologies and management approaches (Pretty & Bharucha, 2014). The Eastern Indo-Gangetic Plains of South Asia host the world’s highest density of rural poor, pervasive yield gaps, and relatively abundant water resources (Bharati, Sharma, & Smakthin, 2016; Jain et al., 2017). They are therefore a global priority for sustainably increasing food production while ensuring that yield gains are accompanied by acceptable social and environmental costs and that long-term viability of the resource base is maintained.

Despite an overall abundance of water resources, water stress is one of the main factors limiting staple crop productivity in the Eastern Indo-Gangetic Plains. Increasingly erratic monsoon-season (*kharif*) precipitation poses a particular threat to the rice-based production systems that dominate the landscape (Turner & Annamalai, 2012). Many farmers in the Eastern Indo-Gangetic Plains apply supplementary ‘life-saving’ irrigation to overcome rainfall deficits during *kharif,* which is enabled by shallow water tables and broad coverage of groundwater irrigation infrastructure. Nevertheless, several studies suggest that irrigation use is typically ‘too little, too late’ and that even resource-poor farmers may improve yield, profitability and resilience with more judicious water use (Kishore, Sharma, & Joshi, 2014). Helping more farmers transition from ‘life-saving’ to ‘productivity-enhancing’ irrigation strategies will therefore be crucial for establishing pro-poor sustainable intensification pathways in the Eastern Indo-Gangetic Plains, particularly in view of contemporary climate variability and progressive change.

In contrast to the Western Indo-Gangetic Plains in India and Pakistan, where groundwater decline jeopardizes sustainability, abstraction in many parts of the Eastern Indo-Gangetic Plains is judged to be well within the limits of safe operating space, with significant scope for increased use (Bharati et al., 2016). Government of Nepal estimates suggest that groundwater use in the region could increase more than fivefold before breeching sustainable abstraction thresholds (Shrestha, Tripathi, & Laudari, 2018). Meanwhile, diesel-powered shallow tubewells are quickly growing in number, whereas investment in large canal infrastructure is diminishing (Bharati et al., 2016). This presents an opportunity to increase productivity-enhancing irrigation use in the Eastern Indo-Gangetic Plains, with the proviso that recurrent monitoring and adaptive management are essential to detect and mitigate early signs of receding groundwater levels. However, studies increasingly recognize that infrastructure development alone is insufficient to achieve optimal use of water resources (Qureshi, Ahmad, & Krupnik, 2015). Shallow tubewell-based irrigation is encouraged or discouraged by farm- and community-scale decisions, along with broader economic and policy incentives (Kishore et al., 2014). Understanding and harnessing these socio-hydraulic dynamics is particularly important in regions like the Eastern Indo-Gangetic Plains that are dominated by smallholder agricultural systems in which farmers face seasonal cash liquidity constraints, the costs of pumping are high, farmers are risk averse, and tenancy arrangements often discourage investments in intensification (Jain et al., 2017; Sugden, 2014).

This study characterizes drivers of shallow tubewell irrigation use to identify development support pathways that are likely to increase irrigation use and climate resilience among rice farmers in Nepal’s component of the Eastern Indo-Gangetic Plains, the Terai – the country’s ‘breadbasket’ lowland region, which runs parallel to its southern border with India. To understand the complexities of socio-hydraulic systems, an interdisciplinary perspective is essential (Massuel et al., 2018). We use a mixed-methods approach to characterize shallow tubewell irrigation infrastructure and farmers’ decision processes around its use for *kharif* rice. Emphasis was placed on diesel-pump-based systems, the dominant form of irrigation in much of the Terai. The Terai is similar in physiography and cropping patterns to the neighbouring Indian states of Bihar and eastern Uttar Pradesh, and also north-eastern Bangladesh, although in Nepal agricultural policies and support programmes are less generous and rural infrastructure is generally underdeveloped (Shah et al., 2009).

## Study area

**Figure 1.**
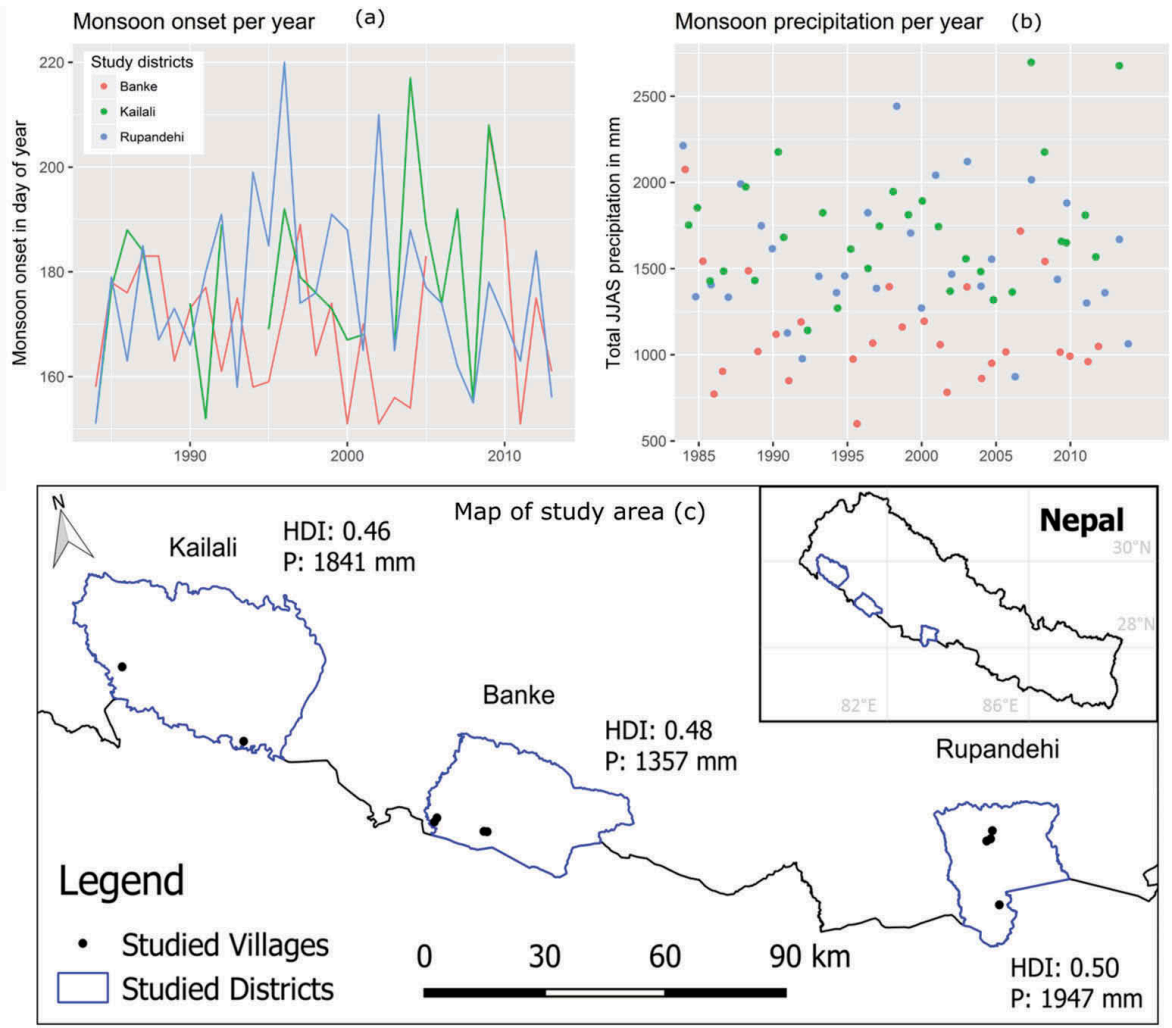
Study area: (a) monsoon onset by district; (b) variability in total June–September precipitation; (c) locations in the Terai of Nepal where household interviews and surveys were conducted.

This study was conducted in three districts of Nepal’s Terai: Rupandehi, Banke and Kailali. These districts span a co-varying gradient of precipitation and wealth (Figure 1). They were chosen in consultation with senior irrigation policymakers who verified the district’s representativeness for the Terai. In each of these districts, around 80% of the precipitation and river discharge occurs from July to October, during the monsoon (Shrestha et al., 2018). Flooding is common during this period. River flows diminish significantly shortly thereafter, hindering the use of canal-based surface water irrigation on a year-round basis (Bharati et al., 2016). A large part of Nepal’s cultivated land area (ca. 40%) is reportedly reached by canal irrigation schemes, but considerably less is actually used. But an estimated 42% of the Terai’s farmers do have access to shallow tubewells, according to the Nepal National Sample Census of Agriculture 2011–2012 (Central Bureau of Statistics, 2012).

The aquifers in the Terai are complex and consist of poorly ordered alluvial sediments that generally become finer towards the south. An unconfined aquifer extends to a depth of around 50 metres below ground level but varies in thickness, sometimes even at the village scale. These unconstrained aquifers may be underlain by a confined aquifer that can extend to a depth of 200 m. (Bonsor et al., 2017). Well yields vary from a few litres per second to more than 10 L/s. Bharati et al. (2016) report an average annual water table depth of 4.6 m across our study areas, with a seasonal fluctuation of about 3 m and a peak in August following recharge from monsoon precipitation. However, no reliable long-term water table data are publicly available, and levels may vary between locations.

## Methods and data sets

### Mixed-methods approach

**Figure 2.**
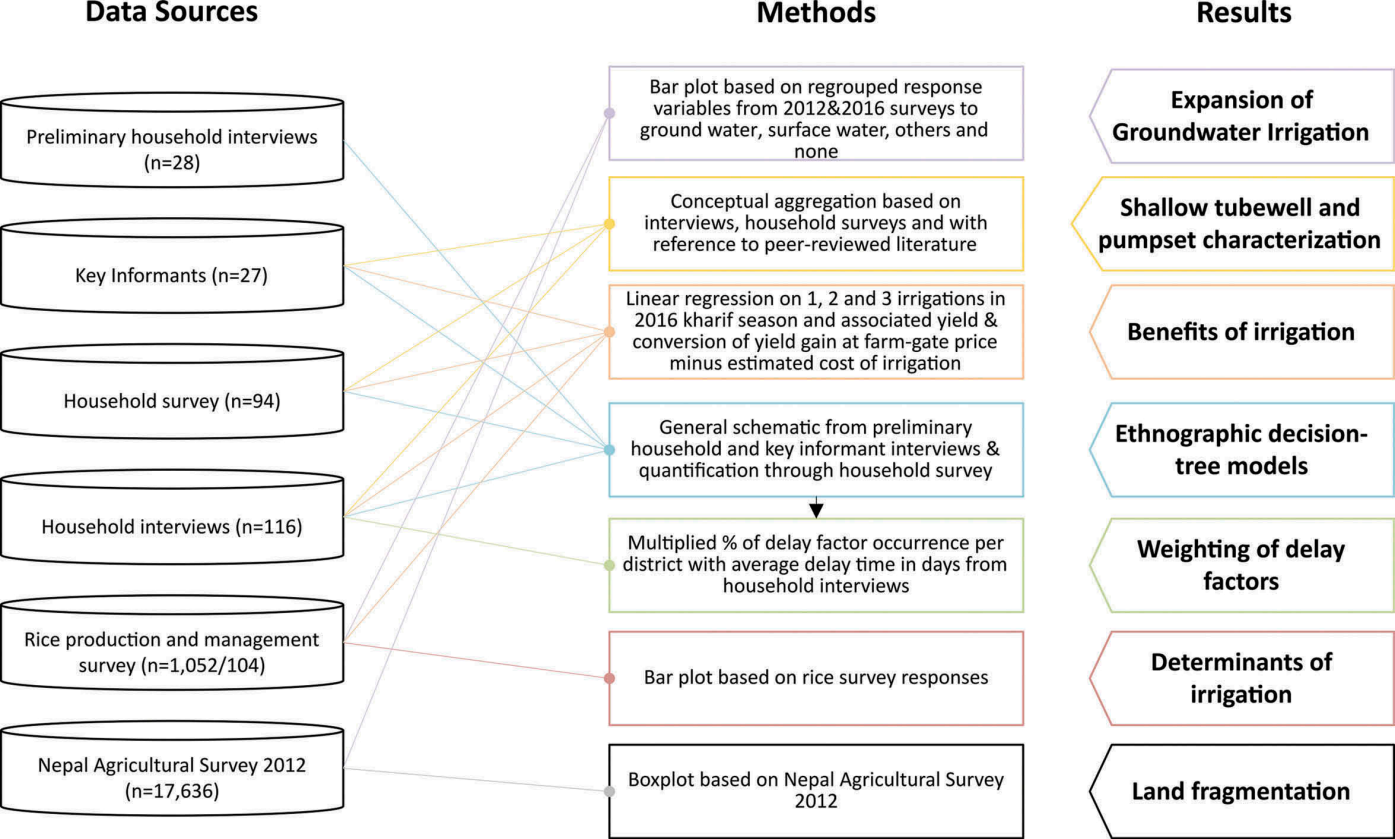
Overview of the data collected, methods employed and results utilized in this study.

To identify drivers of rice irrigation practices in areas where shallow tubewells predominate in the Terai, we employed a mixed-methods approach including semi-structured interviews with farmers, policy makers, and farming communities. These were coupled with household surveys and ethnographic decision-tree models (Roth & Botha, 2009). Interview and survey data were used to define the variables and sequences of decisions represented in the decision-tree models. An overview of data sets, figures and tables, and analytical approach used in their development is presented in Figure 2.

### Site selection

We used time-series estimates of net primary productivity from remote-sensing imagery to identify villages using a range of irrigation practices. Net primary productivity values correlate with crop water uptake (Ciais et al., 2005). Therefore, areas with high temporal variation in agricultural net primary productivity generally have unreliable access to or suboptimally applied irrigation, while areas with low net primary productivity variation can be understood as having less moisture limitation of agricultural productivity.

As a proxy for irrigation, we used MODIS17A3H annual net primary productivity raster data from 2000 to 2014 (500 m^2^ grids) to calculate the standard deviation of net primary productivity for each cell over a 14-year period. Then, we identified areas with a large range of irrigation use surrounding them by assigning each cell the standard deviation of its 3 × 3 neighbourhood. We identified the villages in the upper 10% of variability in access to irrigation surrounding them using a village location database based on a village data shapefile obtained from the Survey Department of the Ministry of Agriculture, Land Management and Cooperative. These villages (four in Rupandehi, three in Banke) were earmarked for our study. Selected locations were cross-checked with agricultural experts in each district to verify the presence of *kharif* rice production and shallow tubewells. This method produced results for Rupandehi and Banke, but the results for Kailali were problematic due to high occurrence of non-cropped areas. Thus, we omitted the neighbourhood calculation for Kailali and selected one village in the upper 10% and one village in the lower 10% of interannual net primary productivity variability.

### Interview and survey data

Data were collected between September and December in 2016. Key informants (27) were chosen from government organizations with a mandate related to irrigation. These included senior officers of the Department of Irrigation, Department of Agriculture and Department of Electricity at the district level. These officers were approached at least twice, once at the beginning of the study and once at the end, to reconfirm responses. At the local level, Village Development Committee secretaries and other local leaders were interviewed on a recurrent basis as questions arose.

At the farm level, semi-structured household interviews (116) and more formal household surveys (94) were conducted. At least 12 household heads were randomly interviewed in each village, covering all sub-village administrative units (blocks). Following interviews, short ad hoc group discussions were often had with neighbours. Data from these sources were used to cross-check previous results and were added to the household interview data set. We also used secondary data from the Nepal Agriculture Survey and the National Census of Agriculture, collected in 2011–12 (Central Bureau of Statistics, 2012), along with data from rice production practice surveys (1052 households) in the Terai collected by the Cereal Systems Initiative for South Asia (CSISA) in 2016 (Paudel, Maharjan, Guerena, Rai, & McDonald, 2017).

### Shallow tubewell irrigation characteristics

To develop the context in which decisions are made and aid interpretation, we used several auxiliary data sets to complement the ethnographic decision-tree models. The spread of shallow tubewells was estimated with data from the Nepal Agriculture Survey (2012) and the CSISA production practice survey, as described earlier. Pricing data and technical specifications for pumps and tubewells were collected from machinery dealers and well drillers. Irrigation costs paid by farmers were estimated through surveys and household interviews. Interview data were triangulated with several key informants and farmers.

**Figure 3.**
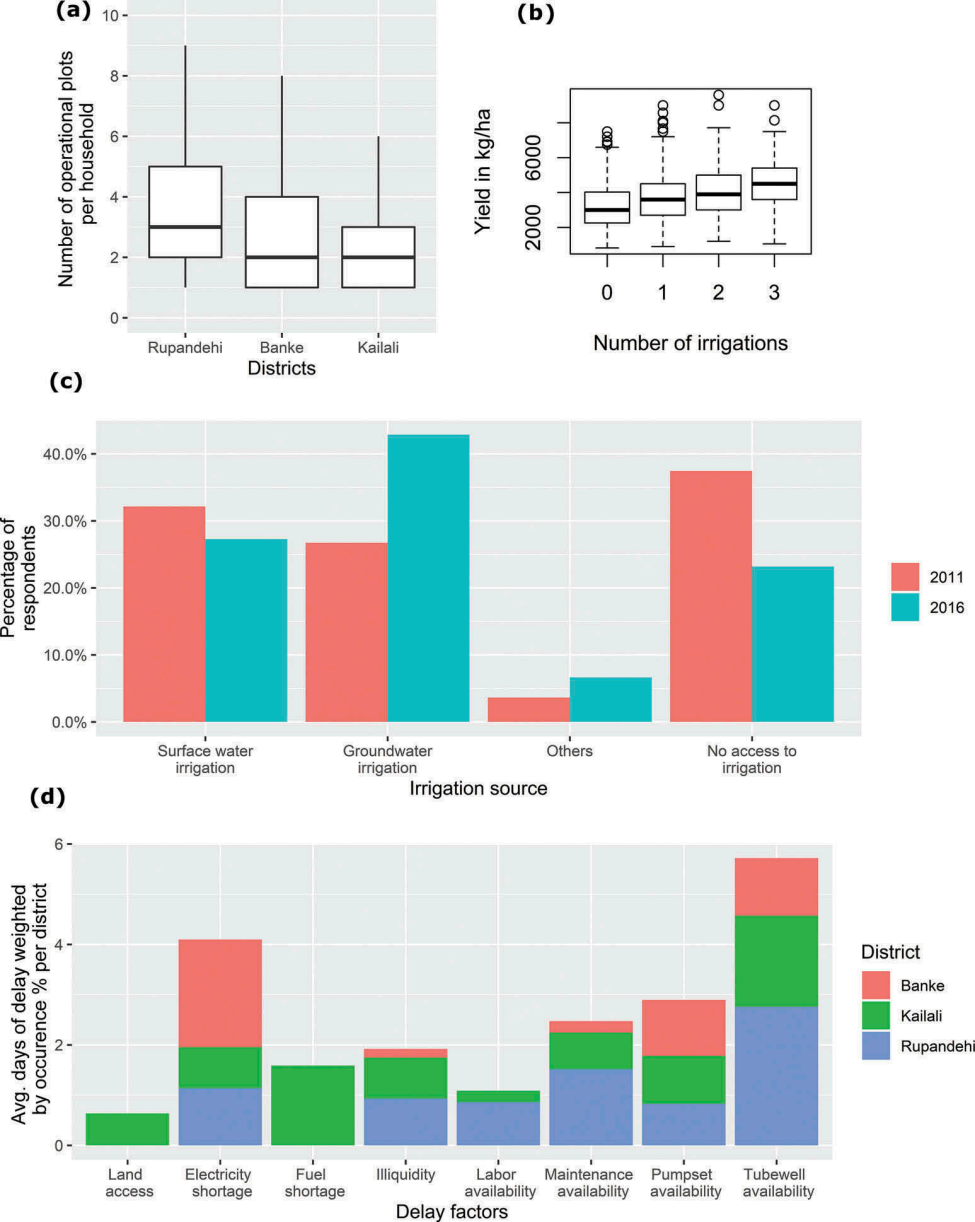
Shallow tubewell irrigation characteristics: (a) land fragmentation in western Terai; (b) yield increase per irrigation (Paudel et al., 2017); (d) relative importance of supplementary irrigation delay factors reported by *kharif*-season rice.

Potential profits from supplementary irrigation applied to *kharif* rice were estimated by combining operational cost data for irrigation from our own surveys with ‘farm gate’ price data for rice from the FAO and the incremental yield benefits of additional irrigation estimated by linear regression from the CSISA production practice survey. An asymptote regression fit would be appropriate, but we lacked sufficient observations of farmers applying more than three irrigations. So we focused on the yield increase associated with one, two and three irrigations, which can be described by a linear fit (Figure 3). The benefit is expected to level off for higher numbers of irrigations, as other inputs such as seed or fertilizer may become limiting factors; it will also vary significantly by year, depending on rainfall patterns. We take the increased profit from additional irrigation (in USD per hectare) to be

(1) (FGP×YI)−(IP×ID×10,000×EF)

where FGP is the farm grate price of rice (USD/kg), YI is the yield increase per irrigation (kg/ha), IP is the irrigation water price (USD/m^3^) translated from hourly rates charged in the field at an assumed discharge of 10 L/s, ID is irrigation depth (m) (Sudhir-Yadav, Humphreys, Kukal, Gill, & Rangarajan, 2011), and EF is water losses of 33%, commonly found in basin irrigation. The SD in yield per hectare translates to USD 18 per hectare using the same assumed irrigation costs. We assumed USD 1 per litre of diesel, USD 3 per hour of pumping as a pump rental price (based on key informants), USD 260 per ton of rice produced (based on FAO information), and 368 kg rice yield increase irrigation per irrigation (based on linear model fit to rice crop cut survey data; coefficient 368.46, standard error 36.34, *p* ≤ 2e-16).

Precipitation information (1984–2013, Figure 1) was drawn from government data from stations in Bhairawa (Rupandehi), Nepalgunj (Banke) and Sandepani (Kailali). The monsoon onset dates used in Figure 1(a) were calculated using the methods of Fitzpatrick, Parker, and Willetts (2016). Human Development Index (HDI) is based on United Nations Development Programme (2014). The land fragmentation figures in Figure 3(a) are based on Central Bureau of Statistics (2012) data.

### Ethnographic decision-tree model construction

Ethnographic decision-tree models are flow charts that aim to capture decision-making processes from the language and concepts respondents use rather than a predefined conceptual framework. They are based on interviews and subsequent cross-validation (Roth & Botha, 2009). They are suitable for representing decision processes of rather homogeneous groups, such as farmers within an agro-ecosystem or entrepreneurs within a city (Roth & Botha, 2009). Our model was based on preliminary unstructured interviews with farmers to identify factors that influence the choice to provide supplementary irrigation to rice. Factors that negatively influence or delay farmers from choosing to provide supplementary irrigation were also identified. Our approach followed Roth and Botha (2009) and was based on evidence that farmers’ general perceptions are sufficiently reliable indicators of local trends and patterns (Banerjee, Chandrasekhar, Duflo, & Jackson, 2018). We first built a generalized decision schematic through key informant interviews with farmers and their perceptions of decision points and factors that influence them. To quantify these relationships, results from the household survey were used to assign each branch of the decision tree a weight reflecting the proportion of farmers making that decision. We then compared the quantifications across districts.

Ethnographic decision-tree models were only constructed with farmers that have access to shallow tubewells for supplementary irrigation. The decision process is initiated by farmer assessments of the need for irrigation and may be reset at any point that sufficient rainfall is received before irrigation has been applied. Similarly, to highlight bottlenecks for the use of supplementary irrigation for *kharif* rice, we represent changes in decision preferences only in the case of irrigation delay, not for situations in which farmers choose to irrigate unencumbered.

The ethnographic decision-tree models have two components: irrigation triggers and delay factors. The first refers to when and under what circumstances farmers decide to apply supplemental irrigation. The CSISA production practice survey (Paudel et al., 2017) indicates that plant (59%), soil (56%), weather (43%) and neighbouring farmers’ practices (31%) play a role. Farmers indicated that disagreements among them on the status of their crops or soil were relatively rare. The terminology used to describe crop and weather tended to be subjective and idiomatic (e.g., ‘plants smile’, ‘clouds look fatter’). Soil moisture was most commonly described in terms of the width of cracks that form as rice fields dry over successive days without floodwater. These were taken as ‘no cracks/hairline cracks’, ‘small cracks’ (up to 2.5 cm wide), and ‘large cracks’ (over 2.5 cm). Since the soil criteria are readily observed by farmers and fairly consistently applied as a management rule, we decided to limit ethnographic decision-tree model quantification to soil conditions to maintain parsimony, reduce translation error and ease comparison between farmers.

Irrigation delay factors are the second component. Shallow tubewell delays are irrigation lags associated with turn-taking as farmers wait to use shared well infrastructure. Energy delays happen when electricity or fuel (depending on the type of pump) is needed but unavailable. Liquidity delays refer to the inability of farmers to pay for energy or rental of a pumpset when required. Pumpset delays are caused when a farmer must borrow or rent a pump. Transport delay refers to the transport of pumps to boreholes in command areas lacking road access. (Moving engines across established fields can damage crops; it is generally only permitted if neighbouring farmers have already irrigated.) Labour delays refer to a dearth of farm labour to oversee irrigation. As irrigation is generally considered a task for men, some households engage relatives or friends if male members are not present. Finally, maintenance delays refer to the time it takes to source spare parts or engage a technician to repair a broken pumpset.

## Results: shallow tubewell irrigation characteristics and use patterns

### Key shallow tubewell irrigation characteristics

Pumpsets deployed for shallow tubewells in the Terai are described in Table 1. Using standards described by Bom, van Raalten, Majundar, Duali, and Majumder (2001), oversized and inefficient pumpsets of 5 horsepower or more are common. Farmers and agricultural machinery dealers differentiate between large pumps (5–10 HP, not easily transportable) and smaller and more portable pumps (3–5 HP). These might seem more suitable for vegetable cultivation. But at a constant discharge, the horsepower requirement of a pump depends on system head (the pressure required to move water through the system). With well yields of ca. 10 L/s, and only a few metres of system head, the smaller pumps are adequate. With increasing system head (e.g., because of several hundred metres of delivery pipes) higher horsepower is warranted, or discharge may decrease.

**Table 1.** Overview of locally available pumpsets.

	Capital expenditure (USD)	Operational expenditure (USD/h)	Power (HP)	Mobility	Popularity of *kharif* rice irrigation	Potential profit from extra irrigation (USD/ha)
Large diesel pumpsets	350–650	1–2 (0.003/m^3^)	5–10	Immobile, transported by bullock cart	High. Regarded as strong and durable. Status symbol.	57
Small diesel pumpsets	250–450	0.3–0.7 (0.014/m^3^)	3–5	Mobile, transported by bicycle	Low. Regarded as weak and easily damaged.	83
Electric pumpsets	150–250	0.07–0.12 (0.042/m^3^)	1.5–2.5	Mobile, transported by bicycle	Medium. Inexpensive and efficient, but depends on unreliable and hard-to-get electricity.	93
Rented pumpsets	0	3–4.5 (0.083/m^3^)	3–10	Depends on rented pumpset	High, but depends on availability, social capital and cash/credit availability.	18

Source: Household interviews and surveys, key informants.

The distribution of small and large pumpsets is nonuniform: 72% of small pumpsets owned by surveyed households were found in Banke District, but only 18% of large pumpsets. The reasons for this disparity are not clear, but household survey respondents said that high mobility of the pumps, a lack of financial liquidity and a relatively high irrigation frequency with lower energy costs are the main drivers of the preference for small equipment. The smaller pumps, primarily imported from China, are perceived as less durable, while the larger pumps, primarily from India, are considered strong and more reliable, although their higher cost and power make them a worse fit for most shallow tubewell infrastructure.

Between 2011 and 2016, groundwater abstraction in the Terai using shallow tubewells increased by approximately 15%, while surface water irrigation decreased by roughly 5% (Figure 3). Data from the CSISA production practices survey also suggest future growth in shallow tubewell infrastructure, with nearly half of the respondents planning to provide supplementary irrigation to *kharif* rice more frequently. In addition, 34% of farmers surveyed indicated their intention to purchase shallow tubewell technologies (e.g., boreholes, pumps, piping) in the next year. This is not surprising given that yield benefits may bring substantial economic benefits (Table 1). Even in a ‘good’ monsoon, clear yield benefits can be expected from increasing supplementary irrigation intensity. The 2016 CSISA survey shows that farmers produced 368 kg/ha more rice, on average, when irrigating a first, second or third time (SE 34 kg); very few farmers irrigated four or more times. The impact of additional irrigations, as well as the timing of irrigation and growing-season weather, are important but beyond the purview of this analysis. Based on current practices in the Terai of Nepal, applying additional irrigation is generally lucrative for pump owners and potentially for pump renters, if rental prices can be reduced.

From the farmer and household survey, as well as key informant interviews, operational aspects emerged as another factor of considerable, and perhaps intensifying, importance to groundwater irrigation. Land fragmentation, measured as the number of operational fields maintained by a farm household, decreases from east to west, with an interquartile range of 2–5 non-adjacent plots per household in Rupandehi, decreasing to 1–3 in the far-western Kailali District (Figure 3). These data indicate that transaction costs for shallow tubewell use are much higher in Rupandehi than in Kailali.

### Shallow tubewell irrigation use patterns

**Figure 4.**
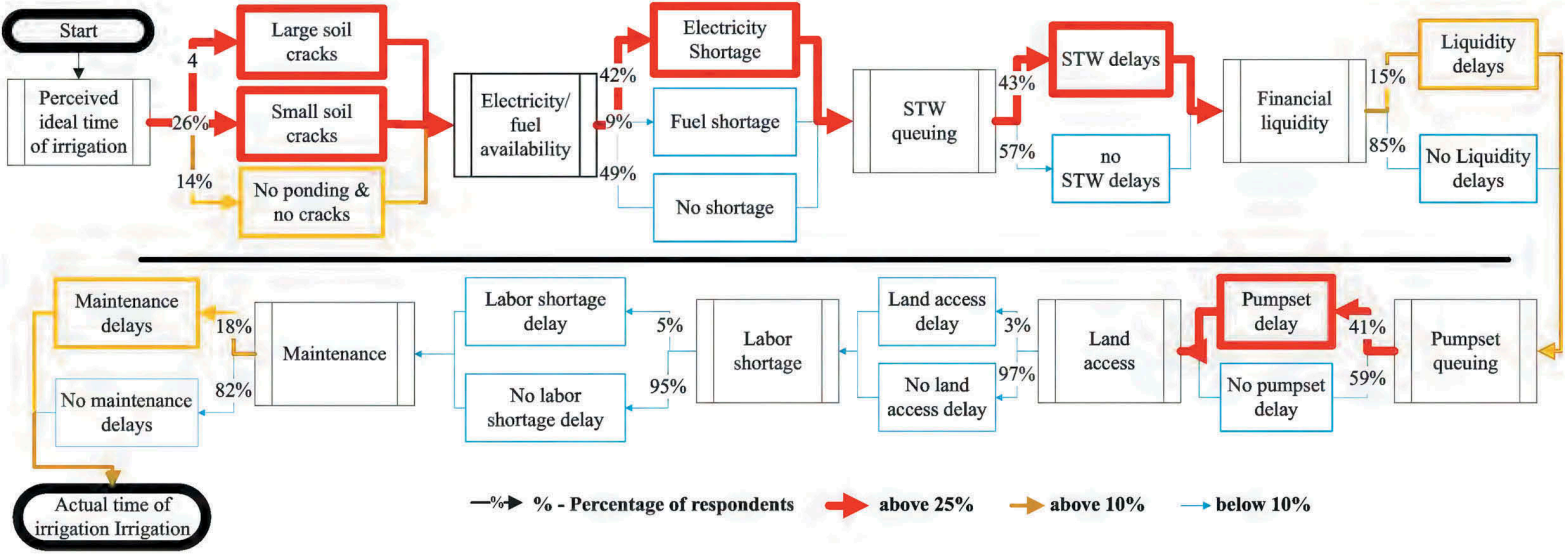
Quantification of ethnographic decision-tree models regarding the decision to use shallow tubewell irrigation, based on household survey results in all three study districts. The decision process can be restarted any time rain occurs.

Most surveyed farmers decide to apply supplementary irrigation to rice in the *kharif* season based on visual observation of soil cracking as drying occurs; they wait until large cracks (over 2.5 cm) are present before irrigating (Figure 4). But criteria differ between districts, with 47% of the farmers in Banke waiting for large cracks, whereas 68% and 71% of farmers wait for large cracks in Rupandehi and Kailali, respectively. Key informant interviews indicated that Banke farmers tend to irrigate earlier because of access to electric pumps and prevalence of small pumpsets, which are less expensive to operate than the large pumpsets mostly found in Rupandehi and Kailali. Our data suggest that most farmers do not recognize the link between delayed irrigation and yield outcomes, as irrigation is often considered as a mechanism to save the crop rather than as a productivity-enhancing investment, and farmers often attribute yield penalties to inferior quality of groundwater compared to rainwater.

Factors influencing farmers’ choice to delay *kharif*-season supplementary irrigation of rice, weighted by their percentage of occurrence and average time of delay, are presented in Figure 4 and Table 2. Pumpset rental and queuing at shallow tubewells are major delay factors in all districts. In Kailali and Rupandehi, maintenance and liquidity constraints cause delays, pointing to problems with the availability of overly expensive and high-horsepower diesel pumpsets, primarily from India. Differences between land access and labour availability reflect higher rates of male labour out-migration in Rupandehi than in other districts (Ministry of Labor and Employment, 2017). Land access also reflects older and harder-to-transport pumpsets being used in Kailali. In Banke, electricity shortages and poor maintenance of pumps are important constraints. Based on these results, we discuss potential and complementary policies and development interventions that can be applied at relevant spatial scales and that could help optimize the use of supplementary irrigation for *kharif*-season rice in the Terai in the face of increasing precipitation uncertainty.

**Table 2.** Irrigation delay factors reported by monsoon-season rice farmers, with average delay and percentage of occurrence in each district.

		Occurrence (%)		
Delay factor	Average delay (days)	Kailali	Banke	Rupandehi
Transportation	7	9	0	0
Electricity shortage	3	27	71	38
Fuel shortage	5	32	0	0
Illiquidity	3	27	6	31
Labour availability	5	5	0	17
Maintenance availability	4	18	6	38
Pumpset rental	3	32	37	28
Queuing at shallow tubewell	4	45	29	69

Nearly half (47%) of all delay pathways pertain to one of the following four pathways: only electricity supply constraints (23%); only shallow tubewell queuing (8%); only pumpset delays (6.7%); and all of the three previous factors together (9%). Once a decision has been made to irrigate, on average farmers reported that total delay (after the decision) amounts to one week, and that irrigation takes place 13 days after the last rainfall event (12.9 days for electricity delays, 10.4 days for queuing delays, 11.3 days for pumpset rental delays, and 15.25 days for all three occurring together).

## Discussion

All three districts seem to follow a similar overall development trajectory of increasingly erratic monsoon rainfall to which farmers respond through shallow tubewell expansion. But late irrigation scheduling and delay factors reduce productivity and production stability. This is reflected in generally long delays for queuing for wells and pumpsets in all districts. On closer inspection, however, precipitation and wealth differences between the districts arguably attune them to differing configurations of the same overall development trajectory. Most notably, Banke receives considerably less rainfall, leading to coordinated government investments in agricultural electrification and thereby incentivizing farmers to invest in electric pumpsets. This is reflected in the finding that unreliability of electricity is more critical in Banke than in the other two districts. Higher moisture stress and familiarity with smaller-horsepower electric pumpsets may also explain the prevalence of smaller-horsepower diesel pumpsets in Banke than in Kailali and Rupandehi.

Kailali and Rupandehi receive similar amounts of precipitation, but Rupandehi is wealthier. Increasing labour shortages (due to structural changes in the economy away from agriculture) and less fuel shortages are arguably a sign of this disparity. Similarly, land access issues persist in Kailali but not in Rupandehi, pointing at a larger use of bullock carts for transporting pumpsets to the field. Financial liquidity appears equally important in Kailali and Rupandehi but less so in Banke. This indicates that more frequent pump rental in Rupandehi and large inefficient pumpsets operated by resource-poorer farmers in Kailali remain a key issue in diesel pump irrigation, in contrast to areas where electric and small and efficient pumpsets are more common.

A single overall strategy to support farmers in responding effectively to monsoon breaks is therefore unlikely to suffice, because sustainable irrigation development needs to cater to contextual factors that vary geographically and even between households within the same community. Furthermore, groundwater development programmes risk widening existing social inequality between users (Wilson, 2002). This calls for pro-poor governance frameworks to ensure that different strata of water users benefit equitably from groundwater development. Recognizing these challenges, we propose three modular support pathways that correspond to nested spatial scales and can be prioritized for investment according to the socio-hydraulic characteristics of the target region. The main goal is to reduce the socio-economic barriers to groundwater use in an equitable and sustainable manner. Lastly, we briefly discuss the current state and potential of electrically powered shallow tubewell use and highlight the need to improve groundwater governance along with increasing use patterns.

As a general caveat, our findings should be contextualized within the longer-term change and development processes in this region. A recent drop in prices for solar panels has convinced many scientists, organizations and governments that solar-powered irrigation systems will be a key transformative technology for rural development in South Asia (Hartung & Pluschke, 2018). Similarly, policy imperatives for improved rural electrification, such as the ambition that Nepal could be the Battery for South Asia and India’s initiative to bring electricity to ‘the last village’, suggest that grid-powered irrigation could plausibly increase in the nearer term, although large uncertainties remain about the pace and extent of rural electrification. The support pathways put forward in this article seek to identify options that are likely to encourage improved water use for diesel-powered irrigation until reliable grid- and solar-powered irrigation become widely available. Improved diesel systems could also be of use where electrically powered irrigation systems remain unfeasible. Understanding farmers’ decision making with regard to current technologies could also contribute to better delineating problems and identifying target groups for scaling up solar and grid-powered irrigation systems. But such analyses remain outside the scope of this article.

### Support pathway 1: efficiency gains at the farm level

Our findings point at two key interventions that could improve the water and energy efficiency of pumpset irrigation at the farm level to encourage farmers to move from ‘life-saving’ to ‘productivity-enhancing’ irrigation of *kharif* rice, one regarding irrigation scheduling and the other regarding operational efficiency.

#### Irrigation scheduling

Rice farmers in the Terai generally use the formation of large soil cracks as a cue for irrigation scheduling. This strategy can dramatically increase the costs of irrigation due to percolation losses from the root zone. Furthermore, allowing soils to dry past 40 kPa water tension can reduce rice yield by 50% in some soil types (Sudhir-Yadav et al., 2011); and large cracks typically form in even drier conditions. In addition to limited knowledge of the relationship between water stress and yield loss, late irrigation scheduling may also reflect farmers’ tendency to be cash-investment averse rather than yield-or-profit-loss averse. In the case of marginal farmers, investment aversion in the Eastern Indo-Gangetic Plains is often linked to unfavourable tenancy agreements (Sugden, 2014). Risk aversion has been observed elsewhere as a limiting factor to the adoption of climate-smart agricultural practices intended to increase resilience, a situation in which both awareness raising and policy measures may be needed to boost uptake (Rai, Bhatta, Acharya, & Bhatta, 2018). Changing farmer’s perceptions of the value and profitability of timely irrigation of *kharif* rice, in tandem with lowering irrigation costs to reduce investment aversion, have been observed to raise crop production and farmers’ income (Qureshi et al., 2015).

Another factor in farmers’ willingness to implement timely scheduling is uncertainty about the occurrence of the next rainfall. In general, the surveyed farmers are understandably reluctant to irrigate if rainfall could occur. Unfortunately, weather forecast information and climate-informed advisories for irrigation have not yet been widely deployed in South Asia. The World Meteorological Organization has acknowledged the need to improve the availability of climate information to better inform decision makers, with specific attention to agriculture. The Global Framework for Climate Services and a number of pilot efforts have developed out of this (Hewitt, Mason, & Walland, 2012). Another approach emerging in agricultural climate services is the sharing of crucial climate data made available by global producing centres with regional climate centres and national hydro-meteorological services. But the challenge is to build scalable decision frameworks that effectively leverage climate services and communicate them successfully to farmers to encourage behavioural change towards cost-effective and risk-reducing irrigation management.

Lastly, farmers mistakenly believe that groundwater is inferior in quality to rainwater. This can be addressed through clearer extension messaging. Scientific knowledge on irrigation scheduling is mainly anchored in assessing crop water requirements (e.g., Sudhir-Yadav et al., 2011), and advisory and decision-support systems may benefit from addressing these findings in their trainings and messaging.

#### Improving operational efficiency

Late irrigation scheduling practices in the Terai are primarily influenced by cash-investment aversion, so lowering operation costs may be an additional strategy to encourage farmers to move from ‘life-saving’ to ‘productivity-enhancing’ irrigation of *kharif* rice. Our results suggest that the use of inappropriately sized pumps reduces operational inefficiency through wasteful energy consumption. Mismatching pumps is a common problem in developing countries, where information on pumpset efficiency is often lacking and the choice of pumps is limited (Bom & van Steenbergen, 1997; G.J. Bom et al., 2001). For example, a crucial efficiency issue is the problem of friction losses in water delivery pipes. After vertical pumping, many farmers only need to use delivery pipes for short horizontal distances, while others may have to convey water over hundreds of metres using lay-flat pipes. Pump power requirements are much lower for the former group, especially with larger-diameter lay-flat pipes.

Several measures can address the prevailing scenario of low pump efficiency. First, simply adjusting engine speed can reduce fuel consumption by more than 50%, from 1–1.5 L/h to 0.5 L/h, while maintaining the same level of discharge (Bom et al., 2001). Second, market availability of energy-efficient and lower-cost small pumpsets can be improved. However, spare-parts availability for small pumpsets is currently insufficient, so this is an important consideration in efforts to increase irrigation efficiency. Third, energy loss to friction in lateral distribution piping can be reduced by making strategic use of gravity flow through lay-flat pipes or elevated tanks with attached delivery pipes. Larger-diameter delivery pipes can also be used to reduce friction loss, though potential trade-offs with the cost of larger pipes requires further investigation. Fourth, local technicians can use better and cheaper shallow tubewell construction techniques to reduce fuel consumption by 30% per unit of water pumped (Gert Jan Bom & van Steenbergen, 1997) – e.g., using mosquito nets as screens to cover pipe suction holes, or cleaning the well borehole after construction to increase inflow.

In general, guidelines on pump choice, irrigation system design and well construction could be made broadly available through trainings, pump dealers, and state extension services, but gaps between existing information and local knowledge of technical systems must also be addressed. Social marketing campaigns could also be aimed at addressing perception biases towards larger pumps, to educate farmers as to the benefits of smaller and more efficient pumpsets. The development of a common database as a clearinghouse of independently measured technical specification for pumps could also help farmers and policy makers with irrigation investment decisions. Without accurate knowledge of the importance of proper pump choice, pump dealers currently have limited capacity to promote energy-efficient solutions. Shopkeepers are happy to sell more expensive and less efficient pumps, and farmers who lack information will continue to purchase them. Our surveys indicated that pump mechanics also recommend less efficient and more costly pumps to farmers because they receive commissions from machinery dealers. Thus, efforts are needed to align the supply of technically sound information on pump efficiency with incentives to promote the use of smaller but ultimately more suitable pumpsets for shallow tubewells and *kharif* rice irrigation in the Terai.

### Support pathway 2: improving community-level water markets

Our data demonstrate that most farmers in the Terai have several small and scattered fields. This is a clear challenge to irrigation coordination, although it also represents an opportunity to share resources with neighbouring farmers through water markets, i.e., users renting out their pumping equipment, their borehole, or both to other users. In all districts, delay factors associated with borrowing pumpsets and queuing for shallow tubewell use are common. Informal water markets with monopolistic pricing schemes erode the profitability of groundwater use for many farmers (Shah et al., 2009; Sugden, 2014). In addition to the well-known price distortions that may contribute to late irrigation scheduling, the observed coordination problems suggest two more factors that limit the efficiency and equitability of groundwater markets where renters could theoretically profit. First, the high land fragmentation increases coordination difficulties, as farmers need to arrange irrigation access in advance with several shallow tubewell owners. Second, farmers with limited financial resources experience financial illiquidity, as they have already invested their available cash in raising and transplanting rice seedlings. The latter situation in particular challenges the development of efficient water markets to overcome moisture constraints in the face of precipitation uncertainty.

Another issue that requires attention is male out-migration from rural areas. In our survey, this is mainly noticeable through the time delays reported by farmers in finding and hiring agricultural labourers in Rupandehi, the district with the largest migration rate (Ministry of Labor and Employment, 2017). Since irrigation of field crops is predominantly a male task, households with migrated men rely on relatives or neighbours to assist, or they need to wait for a male household member to return home after work. More policy attention to the gender context is required as this trend intensifies. While there is support for expanding the role of women as decision makers in agriculture, service provision provides another approach to address this delay factor.

The first pathway requires improving the organizational components of informal water markets to address the prohibitive cost of irrigation. The provision of low-interest financial services to farmers, so they may overcome within-season cash constraints prior to harvest and the sale of rice grain, is likely to be important in efforts to move farmers to ‘productivity-enhancing’ shallow tubewell irrigation use (Bhandari & Pandey, 2006). Lastly, and perhaps most importantly, taking an anticipatory approach and agreeing on terms and conditions for pump rental and irrigation well before the season, instead of when large soil cracks appear, is also likely to be crucial, especially given the organizational difficulties posed by land fragmentation. To this end, seasonal precipitation forecasts deployed through meteorological and extension services could help ‘trigger’ irrigation arrangements and the provision of fuel for pumps well in advance of the months when rainfall deficits could occur. But such forecasts require much skill to be taken seriously by farming communities (Hewitt et al., 2012).

### Support pathway 3: regional investment prioritization – selectively increasing infrastructure density

Turn-taking for shallow tubewells and pumpset rental are major delay factors and therewith inefficiency of the informal water markets in all three surveyed districts. This means that mere access to irrigation is not a sufficient condition for timely irrigation. Better organization at the community level can only reduce delays to a certain extent. For more substantial delay reductions, increasing the density of shallow tubewells becomes crucial in areas where infrastructure is present but insufficient. Targeting criteria can be developed from this perspective so that pumps are prioritized where they are most needed, increasing the return on public investment in irrigation infrastructure. This approach can be employed together with data on aquifer characteristics such that shallow tubewell infrastructure development is also prioritized in areas where higher levels of water abstraction can be sustained.

Initiatives to encourage better use of groundwater resources for irrigation are also likely to require policy action to address land ownership patterns. This is because there are fewer incentives for tenant farmers and landlords to invest in shallow tubewell infrastructure on rented land (Sugden, 2014). Where higher pumpset penetration is desired, awareness raising and opportunities for the private sector to encourage market growth are likely to be crucial. For example, supporting spare-parts markets, assuring sufficient mechanic services and increasing commercial availability of pumpsets were prerequisites for the rapid and transformative growth of tubewell use in nearby Bangladesh (Qureshi et al., 2015). Convincing farmers that bigger pumpsets are not always better, e.g., by highlighting the fuel efficiency of small pumpsets, as well as identifying and promoting smaller pumpsets that require less frequent repair and maintenance, could be critical steps to increase pumpset ownership among smaller and marginal farmers.

### Electric pumps: rural electrification and solar-powered irrigation

Given the large reductions in costs and CO_2_ emissions achievable with electric pumpsets, rural electrification or solar-powered irrigation may be the best long-term solution for the sustainable intensification of *kharif* rice. Against the backdrop of groundwater overexploitation due to de facto free electricity in north-west India, it is important to keep in mind that metering electricity consumption can incentivize farmers to irrigate effectively (Mukherji et al., 2009). But the current rural electricity network in the Terai has significant problems, including frequent power cuts. Even when power is available, voltage fluctuations commonly damage pumpsets. And areas with relatively good access to electricity are especially vulnerable to power cuts and voltage fluctuations because irrigation pumping is often the largest consumer of irrigation during short periods, and often overloads the system. Besides the physical availability of electricity, access is often difficult to secure because the permit system in Nepal is highly political. And while the price of solar irrigation is falling, it still remains prohibitive for many farmers.

The benefit of reliable agricultural electrification in the Terai is expected to be enormous for broader policy goals such as food security, poverty alleviation and climate change adaptation. But despite the potential benefits of expanding the grid to rural areas, officials in Nepal report that electricity for irrigation is not a development priority, as irrigation would only constitute 1–2% of potential new revenue sources, with very high installation costs required to reach widely scattered villages (Nepal Electricity Authority, 2017). Thus, improving the efficiency and reach of the diesel-based shallow tubewells continues to make sense as a near-term development priority.

### Groundwater governance: reiterating the case for sustainable and evidence-based management

Groundwater depletion has become a global concern (Famiglietti, 2014), but the opposite is true in the Eastern Gangetic Plains, including Nepal’s Terai. Nepal’s government considers the Terai’s groundwater resources underdeveloped and estimates that 88% of the groundwater that could be abstracted on a sustainable basis (based on annual recharge) is not utilized, providing ample space for increased groundwater use for productivity-enhancing irrigation. In other words, encouraging productive use of water resources within regionally and locally defined sustainable abstraction boundaries will be a key element in reaching sustainable intensification targets in Nepal’s Terai (Steffen et al., 2015).

Establishing a regional evidence base for groundwater governance in Nepal is difficult because the low-resource and low-technology environment poses great challenges for gathering recurrent monitoring data on the highly complex aquifer systems. But implementing a system that identifies persistent groundwater decline is a good first step; since groundwater dynamics are localized, local countermeasures such as managed aquifer recharge can be implemented. Regionally, such planning could leverage the Ganges Water Machine initiative, which is being used as a conceptual tool for managing the Indo-Gangetic Plain’s complex and erratic hydrological cycle (Bharati et al., 2016; Shah, Ray, & Lele, 2018).

## Conclusions

This study has established and ranked the importance of the key factors influencing the use of shallow tubewells for supplementary irrigation in *kharif-*season rice cultivation in the Terai region of Nepal. In areas where diesel pumps predominate, the factors most limiting shallow tubewell use are poor coordination among water users, delays in pump and tubewell availability, and financial constraints coupled with risk aversion towards cash investment. The electric grid permits the use of lower-cost pumps, and solar-powered irrigation systems could reduce operation costs. Electrification may overcome some of these delay factors, but the grid reaches only a small fraction of fields at present, and solar-powered irrigation is likely to remain beyond the financial means of most farmers in the Terai.

Our work indicates that a multiscalar strategy to encourage diesel-pump-based shallow tubewell irrigation, to move Nepali rice farmers from ‘life-saving’ to ‘productivity-enhancing’ irrigation use, could contribute to climate resilience, higher yields and higher profits. At the farm level, raising awareness of the importance of timely irrigation can be coupled with efforts to increase operational efficiency (e.g., pump maintenance, pump sizing, forecast-based irrigation scheduling) to overcome aversion to cash investments. At the community level, better preparation for irrigation events through organization of water markets before the start of the season could reduce transaction costs and delays during the season itself. At the regional level, government support programmes can target areas where tubewell and pumpset density is not yet high enough to ensure that all farmers have timely access to irrigation through water markets.

## References

[CIT0001] Banerjee, A. V., Chandrasekhar, A., Duflo, E., & Jackson, M. O. (2018). Using gossips to spread information: Theory and evidence from two randomized controlled trials. *The Review of Economic Studies, 86* (6), 2453–2490.

[CIT0002] Bhandari, H., & Pandey, S. (2006). Economics of groundwater irrigation in Nepal: Some farm-level evidences. *Journal of Agricultural and Applied Economics*, 38(1), 185–199.

[CIT0003] Bharati, L., Sharma, B., & Smakthin, V. (2016). *The Ganges River basin: Status and challenges in water, environment and livelihoods*. New York: Routledge.

[CIT0004] Bom, G. J., van Raalten, D., Majundar, S., Duali, R. J., & Majumder, B. N. (2001). Improved fuel efficiency of diesel irrigation pumpsets in India. *Energy for Sustainable Development*, 5(3), 32–40.

[CIT0005] Bom, G. J., & van Steenbergen, F. (1997). Fuel efficiency and inefficiency in private tubewell development. *Energy for Sustainable Development*, 3(5), 46–50.

[CIT0006] Bonsor, H. C., MacDonald, A. M., Ahmed, K. M., Burgess, W. G., Basharat, M., Calow, R. C., … Zahid, A. (2017). Hydrogeological typologies of the Indo-Gangetic basin alluvial aquifer, South Asia. *Hydrogeology Journal*, 25(5), 1377–1406. 32025191 10.1007/s10040-017-1550-z PMC6979522

[CIT0007] Central Bureau of Statistics. (2012). *Nepal: National sample census of agriculture 2011–2012*. Kathmandu, Nepal: National Planning Commission Secretariat, Government of Nepal.

[CIT0008] Ciais, P., Reichstein, M., Viovy, N., Granier, A., Ogee, J., Allard, V., … Valentini, R. (2005). Europe-wide reduction in primary productivity caused by the heat and drought in 2003. *Nature*, 437(7058), 529–533. 16177786 10.1038/nature03972

[CIT0009] Famiglietti, J. S. (2014). The global groundwater crisis. *Nature Climate Change*, 4(11), 945–948.

[CIT0010] Fitzpatrick, R. G. J., Parker, D. J., & Willetts, P. D. (2016). Assessing the level of spatial homogeneity of the agronomic Indian monsoon onset. *Geophysical Research Letters*, 43(22), 11, 867–11, 874.

[CIT0011] Hartung, H., & Pluschke, L. (2018). *The benefit and risks of solar powered irrigation: A global overview*. Rome: FAO & GIZ.

[CIT0012] Hewitt, C., Mason, S., & Walland, D. (2012). The global framework for climate services. *Nature Climate Change*, 2, 831–832.

[CIT0013] Jain, M., Singh, B., Srivastava, A. A. K., Malik, R. K., McDonald, A. J., & Lobell, D. B. (2017). Using satellite data to identify the causes of and potential solutions for yield gaps in India’s Wheat Belt. *Environmental Research Letters*, 12(9), 094011.

[CIT0014] Kishore, A., Sharma, B., & Joshi, P. K. (2014). *Putting agriculture on the takeoff trajectory: Nurturing the seeds of growth in Bihar, India*. New Delhi: International Food Policy Research Institute and International Water Management Institute.

[CIT0015] Massuel, S., Riaux, J., Molle, F., Kuper, M., Ogilvie, A., Collard, A. L., … Barreteau, O. (2018). Inspiring a broader socio-hydrological negotiation approach with interdisciplinary field-based experience. *Water Resources Research*, 54, 2510–2522.

[CIT0016] Ministry of Labor and Employment. (2017). *Labor migration for employment: A status report for Nepal: 2015/2016 & 2016/2017*. Kathmandu, Nepal: Author.

[CIT0017] Mukherji, A., Das, B., Majumdar, N., Nayak, N. C., Sethi, R. R., & Sharma, B. R. (2009). Metering of agricultural power supply in West Bengal, India: Who gains and who loses? *Energy Policy*, 37(12), 5530–5539.

[CIT0018] Nepal Agriculture Survey. (2012). Kathmandu, Nepal: Central Bureau of Statistics.

[CIT0019] Nepal Electricity Authority. (2017). *A year in review*. Kathmandu, Nepal: Author.

[CIT0020] Paudel, G., Maharjan, S., Guerena, D., Rai, A., & McDonald, A. J. (2017). *Nepal rice crop cut & survey data 2016*. Texcoco, Mexico: CIMMYT Research Data & Software Repository Network.

[CIT0021] Pretty, J., & Bharucha, Z. P. (2014). Sustainable intensification in agricultural systems. *Annals of Botany*, 114(8), 1571–1596. 25351192 10.1093/aob/mcu205 PMC4649696

[CIT0022] Qureshi, A. S., Ahmad, Z. U., & Krupnik, T. J. (2015). Moving from resource development to resource management: Problems, prospects and policy recommendations for sustainable groundwater management in Bangladesh. *Water Resources Management*, 29(12), 4269–4283.

[CIT0023] Rai, R. K., Bhatta, L. D., Acharya, U., & Bhatta, A. P. (2018). Assessing climate-resilient agriculture for smallholders. *Environmental Development*, 27, 26–33.

[CIT0024] Roth, H., & Botha, N. (2009). *Using ethnographic decision tree modelling to explore farmers’ decision-making processes: A case study* . 2009 Conference, August 27–28, 2009, Nelson, New Zealand 97157, New Zealand Agricultural and Resource Economics Society.

[CIT0025] Shah, T., Ray, C., & Lele, U. (2018). How to clean up the Ganges? *Science*, 362(6414), 503. 30385549 10.1126/science.aav8261

[CIT0026] Shah, T., Ul Hassan, M., Khattak, M. Z., Banerjee, P. S., Singh, O. P., & Rehman, S. U. (2009). Is irrigation water free? A reality check in the Indo-Gangetic basin. *World Development*, 37(2), 422–434.

[CIT0027] Shrestha, S. R., Tripathi, G. N., & Laudari, D. (2018). Groundwater resources of Nepal: An overview. In A. Mukherjee (Ed.), *Groundwater of South Asia* (pp. 169–193). doi:10.1007/978-981-10-3889-1_11

[CIT0028] Steffen, W., Richardson, K., Rockström, J., Cornell, S. E., Fetzer, I., Bennett, E. M., … Sörlin, S. (2015). Planetary boundaries: Guiding human development on a changing planet. *Science*, 347(6223), 1259855. 25592418 10.1126/science.1259855

[CIT0029] Sudhir-Yadav, Humphreys, E., Kukal, S. S., Gill, G., & Rangarajan, R. (2011). Effect of water management on dry seeded and puddled transplanted rice. *Field Crops Research*, 120(1), 123–132.

[CIT0030] Sugden, F. (2014). *Landlordism, tenants and the groundwater sector: Lessons from Tarai-Madhesh, Nepal*. IWMI research report 62. doi:10.5337/2015.204

[CIT0031] Turner, A. G., & Annamalai, H. (2012). Climate change and the South Asian summer monsoon. *Nature Climate Change*, 2(8), 587–595.

[CIT0032] United Nations Development Programme. (2014). *Nepal human development report 2014: Beyond geography, unlocking human potential*. Kathmandu: Author.

[CIT0033] Wilson, K. (2002). Small cultivators in Bihar and ‘new’ technology: Choice or compulsion? *Economic and Political Weekly*, 37(13), 1229–1238.

